# Sensitivity of an AI method for [^18^F]FDG PET/CT outcome prediction of diffuse large B-cell lymphoma patients to image reconstruction protocols

**DOI:** 10.1186/s13550-023-01036-8

**Published:** 2023-09-28

**Authors:** Maria C. Ferrández, Sandeep S. V. Golla, Jakoba J. Eertink, Bart M. de Vries, Sanne E. Wiegers, Gerben J. C. Zwezerijnen, Simone Pieplenbosch, Louise Schilder, Martijn W. Heymans, Josée M. Zijlstra, Ronald Boellaard

**Affiliations:** 1 Cancer Center Amsterdam, Department of Radiology and Nuclear Medicine, Amsterdam UMC, Vrije Universiteit Amsterdam, Amsterdam, The Netherlands; 2 Cancer Center Amsterdam, Imaging and Biomarkers, Amsterdam, The Netherlands; 3 Cancer Center Amsterdam, Department of Hematology, Amsterdam UMC, Vrije Universiteit Amsterdam, Amsterdam, The Netherlands; 4 Department of Internal Medicine, Amstelland Hospital, Amstelveen, The Netherlands; 5 Department of Epidemiology and Data Science, Amsterdam Public Health Research Institute, Amsterdam UMC, Vrije Universiteit Amsterdam, Amsterdam, The Netherlands; 6 Amsterdam Public Health Research Institute, Methodology, Amsterdam, The Netherlands

**Keywords:** Diffuse large B-cell lymphoma, PET, Convolutional neural networks, Reconstruction

## Abstract

**Background:**

Convolutional neural networks (CNNs), applied to baseline [^18^F]-FDG PET/CT maximum intensity projections (MIPs), show potential for treatment outcome prediction in diffuse large B-cell lymphoma (DLBCL). The aim of this study is to investigate the robustness of CNN predictions to different image reconstruction protocols. Baseline [^18^F]FDG PET/CT scans were collected from 20 DLBCL patients. EARL1, EARL2 and high-resolution (HR) protocols were applied per scan, generating three images with different image qualities. Image-based transformation was applied by blurring EARL2 and HR images to generate EARL1 compliant images using a Gaussian filter of 5 and 7 mm, respectively. MIPs were generated for each of the reconstructions, before and after image transformation. An in-house developed CNN predicted the probability of tumor progression within 2 years for each MIP. The difference in probabilities per patient was then calculated between both EARL2 and HR with respect to EARL1 (delta probabilities or ΔP). We compared these to the probabilities obtained after aligning the data with ComBat using the difference in median and interquartile range (IQR).

**Results:**

CNN probabilities were found to be sensitive to different reconstruction protocols (EARL2 ΔP: median = 0.09, interquartile range (IQR) = [0.06, 0.10] and HR ΔP: median = 0.1, IQR = [0.08, 0.16]). Moreover, higher resolution images (EARL2 and HR) led to higher probability values. After image-based and ComBat transformation, an improved agreement of CNN probabilities among reconstructions was found for all patients. This agreement was slightly better after image-based transformation (transformed EARL2 ΔP: median = 0.022, IQR = [0.01, 0.02] and transformed HR ΔP: median = 0.029, IQR = [0.01, 0.03]).

**Conclusion:**

Our CNN-based outcome predictions are affected by the applied reconstruction protocols, yet in a predictable manner. Image-based harmonization is a suitable approach to harmonize CNN predictions across image reconstruction protocols.

**Supplementary Information:**

The online version contains supplementary material available at 10.1186/s13550-023-01036-8.

## Background

Diffuse large B-cell lymphoma (DLBCL) is the most common subtype of non-Hodgkin lymphoma, accounting for 30 to 40% of all cases [1]. [^18^F]-Fluorodeoxyglucose ([^18^F]FDG) positron emission tomography (PET) in combination with computed tomography (CT) imaging is widely used for diagnosis, staging, prognosis, prediction and response monitoring in DLBCL patients [2]. Different metrics such as metabolic tumor volume (MTV), standard uptake value (SUV), dissemination and textural features are extracted from these images which provide insight into the tumor characteristics. Baseline MTV has proven to be a strong prognostic factor for tumor progression in lymphoma, alongside with disease dissemination features [3–5]. In order to extract these features, the tumor needs to be delineated. This task is time-consuming, user dependent and suffers from inter and intra reader variability. These limitations could be overcome with the help of artificial intelligence (AI). In oncology, AI models are already being assessed for the automation of many different tasks such as delineation and segmentation of lesions with outstanding results [6–9]. An even further step is to use AI to extract complex features directly from the PET images and predict disease progression without prior lesion segmentation*.* Convolutional neural networks (CNNs) are currently being investigated for this purpose and were found to have potential for treatment outcome prediction in DLBCL [10, 11].

Technical aspects of PET imaging, which include but are not limited to image acquisition and reconstruction settings, should be taken into account when analyzing PET scans as these may have an impact on the derived metrics [12, 13]. ComBat harmonization can be used to reduce the variability generated by some of these technical aspects [14]. To accomplish this, ComBat standardizes the means and the variances across the batches of data derived from different scanners/protocols [15]. In a previous study, ComBat was partially able to reduce reconstruction-dependent MTV variability [12]. Kaalep et al. [16] addressed reconstruction-related variability by altering the images instead of the data. They applied a Gaussian filter to scans obtained from European Association of Nuclear Medicine Research Ltd. (EARL) harmonization standards 2. This filter was used to ‘blur’ the images, mimicking the resolution of the EARL1 scans.

We recently developed a CNN to predict the probability of 2-year time to progression (TTP) using maximum intensity projections (MIP) of [^18^F]FDG PET/CT baseline scans of DLBCL patients [11]. The CNN achieved an area under the curve of 0.72 in the internal validation and of 0.74 in the external validation. The model was, however, trained on retrospective data from the HOVON84 trial [17]. These data were predominantly reconstructed using EARL1 reconstruction settings. With the introduction of newer and state-of-the-art PET systems, higher spatial resolution images can be achieved. Thus, multicenter PET studies will, most probable, involve images generated by different systems, hence presenting different image qualities, among other aspects. As with SUV and MTV, PET reconstruction settings may also have an impact on PET-based CNN performance. A recent study showed that PET images derived from block sequential regularized expectation maximization reconstruction yielded a better CNN performance than ordered subset expectation maximization reconstruction for the detection of pulmonary lesions [18]. Besides these recent findings, little is known about how PET-based classification CNNs are affected by differences in image quality, for example, when sites use PET images compliant with EARL standards 1 and 2 or images reconstructed with locally preferred clinical protocol [16, 19].

In this study, we assessed the sensitivity of outcome predictions provided by our recently developed CNN to different reconstruction protocols, as we did in a previous study for the assessment of MTV [12]. Furthermore, we assessed the ability of image-based transformations reported by Kaalep et al. [16] to generate harmonized predicted probabilities and we compared those to ComBat-transformed probabilities.

## Methods

### Study population

For the analysis, we used baseline [^18^F]FDG PET/CT scans from 20 DLBCL patients. From these, 13 patients had been scanned at the Amsterdam UMC and were retrospectively obtained from medical records, with a waiver for informed consent from the Medical Ethics Review Committee of Amsterdam UMC, location VUmc. This study was registered as IRB2018.029. The other seven patients were recruited and scanned at the outpatient clinic of the department of Hematology of the Amsterdam UMC, location VUmc (IRB2019.278) with a waiver for informed consent from the Medical Ethics Review Committee of Amsterdam UMC, location VUmc. Patients included in these trials required to be 18 years or older and have at least one tumor with a diameter of 3 cm or more. Patients with metal implants, multiple malignancies, who had undergone chemotherapy in the past 4 weeks or who were pregnant/lactating were excluded from the trials.

### Image acquisition

Patients scans were performed on two EARL-accredited Philips scanners, Ingenuity TF PET/CT and Vereos PET/CT (Philips Healthcare, Cleveland, USA), with BLOB-OS-TF reconstruction method and an [^18^F]FDG uptake time of 60 min. PET studies were performed in conformity with EANM recommendation using a bed scan duration of 2 min. The mean injected activity was 264.12 megabecquerels (MBq).

### Quality control

Quality control (QC) of baseline [^18^F]FDG PET/CT scans was performed following the criteria described by the EANM guidelines: Eligible scans should hold a liver mean standardized uptake value (SUVmean) between 1.3 and 3.0, and the plasma glucose should not surpass 11 mmol/L [2]. Scans were also excluded during the QC if they were incomplete, total image activity (MBq) was not between 50 and 80% of the total injected FDG activity and/or any DICOM data was missing. In this study, no scan was excluded as all criteria described by the EANM guidelines were met.

### Image processing

**Fig. 1:**
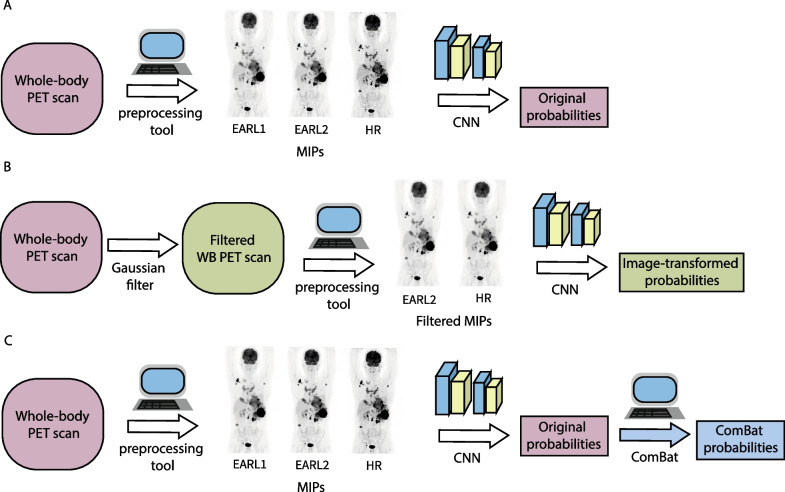
Workflow overview. **A** Generation of original probabilities from whole-body PET scans. The MIPs are generated from the PET scan through the preprocessing tool. The CNN is then used to predict 2-year TTP probabilities. This is done for each of the 3 reconstructed images for all patients. **B** Generation of image-transformed probabilities from filtered whole-body PET scans. A Gaussian filter is applied to the EARL2 and HR scans to obtain images that resemble EARL1-compliant images. The preprocessing tool is used to generate the MIPs from the transformed scans and the CNN is then used to predict the corresponding 2-year TTP probabilities. **C** Generation of ComBat probabilities from whole-body PET scans. To obtain the ComBat-transformed probabilities, ComBat is applied to the generated original probabilities

Three different reconstructions protocols were used to derive the scans: following EARL1 standards (EARL1 reconstruction), following EARL2 standards (EARL2 reconstruction) and a third reconstruction which followed locally clinically preferred protocols (high resolution or HR reconstruction). EARL2 standards were established by introducing a resolution modelling algorithm, point spread function (PSF), to the initial EARL1 standards [16]. The use of PSF improves image resolution and contrast [20]. The main difference between the HR and the EARL reconstructions is that HR introduced a pixel spacing of 2 mm instead of 4 mm to achieve a higher spatial resolution. Table 1 contains a summary of the parameters related to the reconstruction algorithms used in this study. An overview of the workflow followed in this study can be found in Fig. 1A–C.

**Table 1:** Summary of parameters for each of the reconstruction protocols

Method	Series description	Pixel spacing (mm)	Slice thickness (mm)	Reconstruction method	Manufacturer’s model name
EARL1	[WBA_CTAC]-Body	4 × 4 × 4	4	BLOB-OS-TF	Ingenuity TF PET/CT Vereos PET/CT
EARL2	[WBA_CTAC_PSF]-Body	4 × 4 × 4	4	BLOB-OS-TF	Ingenuity TF PET/CT Vereos PET/CT
HR	[HN_CTAC_2mm]-Body	2 × 2 × 2	2	BLOB-OS-TF	Ingenuity TF PET/CT Vereos PET/CT

**Fig. 2:**
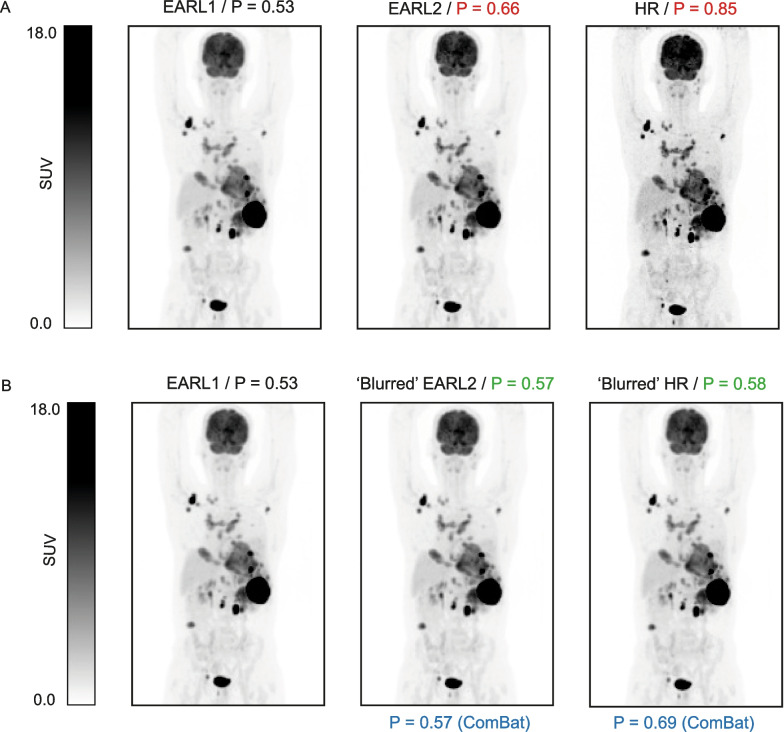
MIP images for the same patient for the three reconstruction protocols. (**A**) MIP images with their corresponding CNN predictions (P). From left to right: EARL1, EARL2 and HR. (**B**) MIP images after image-based transformation (except for EARL1). From left to right: EARL1, EARL2 and HR. Predictions from the original MIPs are shown in red, from the transformed or ‘blurred’ MIPs in green and after ComBat transformation in blue

For each scan and reconstruction, MIPs were generated using an in-house developed preprocessing tool. This tool converted scans into coronal and sagittal MIPs with size 275 × 200 × 1 and pixel size of 4 × 4 mm. Furthermore, the brain was removed from each of the MIPs with the aim of providing greater consistency across the dataset since not all scans fully included the head. More details about this process can be found in our previous study [11]. Example MIPs for each reconstruction are given in Fig. 2A for one of the patients, illustrating the visual difference in spatial resolution between the various reconstructions.

### Harmonization

Two different harmonization techniques were used in this study to align the CNN predicted probabilities between the three different reconstructions: EARL1, EARL2 and HR. We implemented an image-based transformation to change the resolution of our EARL2 and HR 3D PET images to resemble EARL1. This was previously done by Kaalep et al. [16] where they used a Gaussian filter to convert EARL2 compliant PET data to EARL1 compliant images. Moreover, we implemented a data transformation using ComBat to align the CNN probabilities of the EARL2 and HR images to those of EARL1. ComBat was previously reported to reduce the variability for PET-extracted MTV values from differently reconstructed images [12].

*Image-based harmonization*. The ACCURATE software was used to perform the image-based transformation of the EARL2 and HR 3D PET images in order to match image qualities (in particular spatial resolution) with that of EARL1 reconstructions [21]. A Gaussian filter (full width at half maximum, FWHM) of 5 mm was applied to the EARL2 3D PET images and of 7 mm to the HR images to match the spatial resolution to that of EARL1 reconstruction [22]. An overview of the workflow followed to generate the image-transformed probabilities can be found in Fig. 1B.

*ComBat harmonization*. ComBat was applied to the CNN probabilities yielded by the non-transformed EARL1, EARL2 and HR scans. We used ComBat to provide continuity with a previous study on combatting MTV variability [12]. Herein, ComBat was used to align the mean and the standard deviation of the probabilities obtained from HR and EARL2 MIPs to those of EARL1. ComBat was applied using R version 4.0.5 on the code provided by Fortin et al*.* [23]. A detailed explanation about ComBat is given in Additional file 1. An overview of the workflow followed to generate the ComBat-transformed probabilities can be found in Fig. 1C.

### Convolutional neural network

A previously developed CNN to predict the probability of 2-year TTP in DLBCL patients from MIP images is utilized in this study [11]. The CNN was trained on a dataset of 296 MIPs derived from DLBCL baseline scans. These data were predominantly collected on older generation of PET/CT systems using image reconstructions that were mostly consistent with EARL1 reconstruction protocols. The model was trained following a fivefold cross validation and further validated on an external dataset.

The CNN consists of two branches which analyze both coronal and sagittal MIPs through a series of convolutional and max pooling layer and, concatenates with a final fully connected layer. Technical details about the cross validation and the design of the CNN can be found in [11]. The output is a binary prediction given by the probability of TTP longer than 2 years (TTP0) or TTP shorter than 2 years (TTP1), where TTP1 indicates an increased risk of tumor progression or recurrence for the patient. TTP0 may indicate absence of tumor progression or absence of recurrence. In this study we used the CNN to predict TTP1 from the EARL1, EARL2 and HR reconstructions and assessed the impact on those probabilities after image-based and ComBat transformations.

### Statistical analysis

A probability per patient is obtained for each of the three different reconstructions. A non-parametrical statistical hypothesis test, Wilcoxon signed-rank test, was used to compare probabilities between the reconstructions before and after transformation, (image-based and ComBat). The probability difference (delta probabilities or ∆P) was calculated for both EARL2 and HR with respect to EARL1. For EARL2 and HR reconstructions, the median and the interquartile range (IQR) of ∆P were calculated for both scenarios; before and after transformations. Moreover, to estimate the strength of the association between probabilities we used regression analyses and Bland–Altman plot.

## Results

### Patients characteristics

There were a total of 20 patients included in this study. A summary of patients characteristics relevant to the study is given in Table 2.

**Table 2:** Patients characteristics

Patients characteristics	
Sex (N)	
Male (%)	5 (25%)
Female (%)	15 (75%)
Weight (kg)	
Mean (min–max)	77.35 (54–103)
Height (cm)	
Mean (min–max)	176.4 (160–190)
Injected activity (MBq)	
Mean (min–max)	264.12 (164.87–367.48)

### CNN probabilities

Wilcoxon signed-rank test was used to compare paired probabilities, with EARL1 as the reference reconstruction. Statistical differences were found before transformation between EARL1 and HR (p value lower than 0.05). These differences were considerably decreased when comparing to probabilities after both image and ComBat transformation. The resulting p values per comparison can be found in Table 3.

**Table 3:** *p* values from nonparametric Wilcoxon signed-rank test for comparing probabilities between reconstruction with EARL1 as reference

	EARL2-EARL1: *p* value	HR-EARL1: *p* value
Original values	0.055	0.012*
Image-based transformation	0.41	0.79
ComBat transformation	0.88	0.85

Statistical differences (*p* value < 0.05) shown with an asterisk (*)

**Fig. 3:**
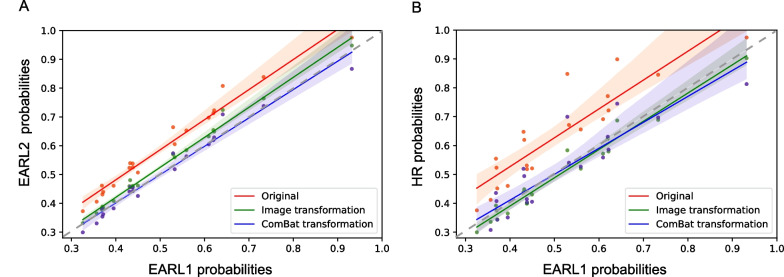
Regression lines for CNN probabilities with EARL1 as reference. **A** EARL2 original probabilities (red), EARL2 probabilities after image-based transformation (green) and EARL2 probabilities after ComBat transformation (blue) compared to EARL1. **B** HR original probabilities (red), HR probabilities after image-based transformation (green) and HR probabilities after ComBat transformation (blue) compared to EARL1. The probability values are closer to the line of identity (gray-dashed line) for both EARL2 and HR values after both transformations

**Fig. 4:**
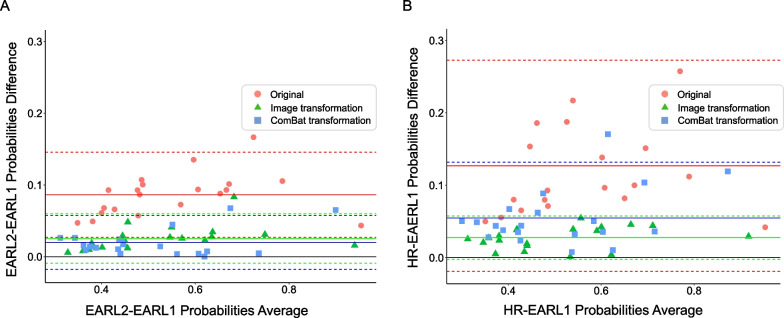
Bland–Altman plots. **A** Bland–Altman plot for EARL2 and EARL1 probabilities, before transformation (in red) and after both image (in green) and ComBat transformation (in blue). **B** Bland–Altman plot for HR and EARL1 probabilities, before transformation (in red) and after both image (in green) and ComBat transformation (in blue)

EARL1 probabilities were generally lower compared to EARL2 and HR probabilities (EARL1 probabilities: median = 0.47 and IQR = [0.38, 0.61], EARL2: median = 0.56 and IQR = [0.46, 0.7], HR: median = 0.64 and IQR = [0.53, 0.74]). An example of the three different reconstructions with their corresponding CNN probabilities can be found in Fig. 2A. The MIP shown in Fig. 2A is an extreme example of how these variations look; EARL1 probabilities are the lowest and HR probability the highest, with a notable difference between them. Table 4 shows the mean differences between reconstructions. The differences were greater between EARL1 and HR than between EARL1 and EARL2 probabilities. Overall, for every patient, an increase in the image resolution led to an increase in the probability values. In the regression plots in Fig. 3 (A), most of the points in red (i.e., original probabilities) are located on a straight line close or slightly above the regression lines. This indicates that the relationship between the probability values generated from different reconstructions is mostly linear; thus, there is a predictable change in the probabilities. This also holds for Fig. 3 (B), although points appear more dispersed in comparison, since the differences from EARL1 to HR probabilities are bigger. From the Bland–Altman plots (Fig. 4), we can derive that differences between probabilities become bigger for higher probability values in both cases, with steeper differences for HR-EARL1 probabilities (Fig. 4 B), suggesting that the differences can be corrected with a scaling factor, as we can also understand from the scatter or correlation plots.

**Table 4:** Overall differences in probabilities (ΔP) between reconstructions with EARL1 as reference

	EARL2 ∆P: Median (IQ)	HR ∆P: Median (IQ)
Original values	0.09 (0.06, 0.10)	0.10 (0.08, 0.16)
Image-based transformation	0.02 (0.01, 0.03)	0.03 (0.01, 0.03)
ComBat transformation	0.02 (0.01, 0.03)	0.04 (0.03, 0.06)

### Image-based transformation

After image-based transformation, the ΔP were considerably decreased and the CNN probabilities showed an improved agreement between reconstructions (Table 4). This is illustrated in Fig. 3 where the regression line of the transformed probabilities values is closer to the line of identity compared to the regression line of the original values. The Bland–Altman plots in Fig. 4 show an improved agreement of the probabilities after image transformation (green data points). Probability differences are constant as the probability average increases for both cases, EARL2-EARL1 and HR-EARL1 probabilities. In Fig. 2B the same example as in Fig. 2A is shown with the MIPs after image-based transformation and their corresponding probabilities. The image-transformed probabilities (shown in green) are considerably decreased compared to the original probabilities indicating an improved alignment.

### ComBat transformation

A similar trend was observed for the ComBat-transformed probabilities. The regression line for the Combat-transformed values is shown in Fig. 3, together with the regression line for the image-transformed values. An improved agreement of the probabilities after ComBat transformation is shown in Fig. 4. For HR-EARL1 probabilities (Fig. 4B), the probability differences (in blue) are larger for higher probabilities average. These differences are less pronounced compared to the original probabilities (in red), which indicates a slightly improved alignment of the probabilities, although not as good as the image-transformed probabilities.

The ΔP was very similar for both image-based and ComBat transformations. However, ComBat resulted in a slightly worsened alignment of the probabilities for the HR reconstruction (Fig. 2B), where there is a greater variability given by the ΔP IQ and also illustrated in Fig. 3B.

## Discussion

The aim of this study was to assess the sensitivity of a CNN model to PET images derived from different reconstruction protocols. The CNN is applied to MIP images generated from [^18^F]FDG PET/CT baseline scans and used to predict the probability of 2-year TTP in DLBCL patients. This model has been internally and externally validated in a previous study [11]. Although CNNs show potential to improve the current state of prognosis in lymphoma [9, 10, 24], there is still an acknowledged need to develop robust and reproducible prognostic markers to eventually attain their clinical implementation [25–28].

In this study, we found considerable differences between the resulting probabilities depending on the reconstruction protocol. For EARL1 images, the probabilities tend to be lower compared to the EARL2 and HR images. The probabilities generated from the HR images, which have the highest resolution in this study, were generally higher across the 20 patients. Overall, we observed that the CNN probabilities were affected by the image resolution in a predictable manner (higher probability values at higher resolution images, i.e., EARL2 and HR). The impact of reconstruction protocols on PET-derived measurements has been assessed in previous studies. SUVmax, SUVpeak, MTV and multiple textural features have all been found to be sensitive to changes in reconstruction protocols [12, 19, 20, 29]. In our previous study, we mitigated the variability among MTV values from images reconstructed with different protocols by harmonizing the data using ComBat [12]. Herein, we assessed the implementation of ComBat for the harmonization of the CNN probabilities and compared it to an image-based transformation. An improved agreement between the CNN probabilities was achieved by both transformations. The image-based transformation was best in aligning the values for the HR reconstruction. These results indicate that transforming the images beforehand could aid in the development of a robust and reliable metric for prognosis and, moreover, facilitate the implementation of CNN models for treatment outcome assessment. When using differently reconstructed images without any transformation, large differences in estimated probabilities may occur (Fig. 2) that can potentially affect clinical decision making. Therefore, reliable predictions using the CNN can only be made when information on the applied acquisition and reconstruction settings are available. In this way the appropriate image transformation can be applied such that the spatial resolution required by the CNN (i.e., conform EARL1) is obtained assuring reproducible estimation of probabilities. A possible limitation of our study is the small number of subjects. However, by performing three different reconstructions protocols for each scan, we were able to directly compare the impact of image quality on CNN-based predictions and allowing us to perform a head-to-head comparison of these probabilities. We observed very clear relationships among the differently obtained probabilities; a higher image resolution led to higher predicted probabilities in a generally linear manner. Moreover, despite the small number of subjects, we believe that we convincingly showed that image transformations can be successfully applied to mitigate reconstruction effects. Another limitation could be that transformations can only be applied when the actual image spatial resolution is known. In most of the cases this can be derived from the DICOM header information, providing details on voxel size and image reconstruction settings. Yet, when using anonymized images, this information is sometimes missing and, therefore, future work will focus on automatically assessing the effective spatial resolution of reconstructed PET data, e.g., using the CNN approach proposed by Pfaehler et al.[30].

In this study, the treatment outcome of the patients is unknown as for this technical validation study the ethics review board demanded use of fully anonymized datasets. Although the CNN was trained with a dataset consisting of mainly EARL1 reconstructed images, and therefore, the correct predictions could be seen as the ones generated by the EARL1 images, we cannot assume such thing without knowing the final outcome of the patients involved. Nevertheless, it is important to understand that the aim of this study was not to assess which predictions were correct, but whether the use of differently reconstructed images would lead to differences in the generated predictions. In this scenario, a higher probability does not necessarily mean a better prediction. The fact that images with higher resolution (i.e., HR reconstruction), thus, better image quality, lead to higher predictions could be due to sharper details and higher intensity contrast in these images. PET studies usually collect data from various centers and thus gathering images with different image qualities. Hence, there is a need to generate tools/methods that can harmonize images and ensure reproducibility.

The architecture of the model and the characteristics of the training data heavily affect the behavior and performance of the CNN. Therefore, the findings of this study cannot be extended to other CNNs [31]. Nevertheless, this study demonstrates that the CNN reported in [11] for the prediction of 2-year TTP in DLBCL patients is a potential prognostic tool which can be adapted to differently reconstructed images. Even though other technical aspects should also be analyzed, this is an important first step toward the use of this model in multicenter studies and/or to translate the tool for data collected using the updated EARL2 standards.

### Conclusion

The predicted probabilities of a previously developed CNN are affected by the applied reconstruction protocol, yet in a predictable manner; higher resolution images (i.e., EARL2 and HR) resulted in higher probability values. After ComBat and image-based transformation, the EARL2 and HR probabilities were closely aligned with EARL1 probabilities. The image-based transformation mitigated the differences slightly better. These findings suggest that image-based transformation is a suitable approach for harmonizing the predictions of this particular model across image reconstruction protocols.

### Supplementary Information

**Additional file 1.** Supplementary Material.

## Data Availability

The datasets generated during and/or analyzed during the current study are available from the corresponding author on reasonable request.

## Abbreviations

DLBCL: Diffuse large B-cell lymphoma
[^18^F]FDG: [^18^F]-Fluorodeoxyglucose
PET: Positron emission tomography
CT: Computed tomography
MTV: Metabolic tumor volume
SUV: Standardized uptake value
Dmax: Maximum distance between two lesions
AI: Artificial intelligence
CNN: Convolutional neural networks
EARL: European Association of Nuclear Medicine (EANM) EANM Research Ltd.
TTP: Time to Progression
MIP: Maximum intensity projections
HR: High resolution
PSF: Point spread function
QC: Quality control
FWHM: Full width at half maximum
SUVmean: Liver mean standardized uptake value
IQR: Interquartile range
