# Supplementary material for: Sensitivity of an AI method for [18F]FDG PET/CT outcome prediction of diffuse large B-cell lymphoma patients to image reconstruction protocols
Source: EJNMMI Res. 2023 Sep 28;13:88. doi: 10.1186/s13550-023-01036-8 (PMC10533444; doi:10.1186/s13550-023-01036-8)
Supplement: Supplementary file 1 — Additional file 1. Supplementary Material. [file 13550_2023_1036_MOESM1_ESM.docx]

**Title**: Sensitivity of an AI method for [^18^F]FDG PET/CT outcome prediction of Diffuse large B-cell lymphoma patients to image reconstruction protocols.

**Authors and Affiliations:** Maria C. Ferrández^1,2^, Sandeep S. V. Golla^1,2^, Jakoba J. Eertink^2,3^, Bart M. de Vries^1,2^, Sanne E. Wiegers^1,2^, Gerben J. C. Zwezerijnen^1,2^, Simone Pieplenbosch^2,3^, Louise Schilder^4^, Martijn W. Heymans^5,6^, Josée M. Zijlstra^2,3^ and Ronald Boellaard^1,2^

^1^Cancer Center Amsterdam, Department of Radiology and Nuclear Medicine, Amsterdam UMC, Vrije Universiteit Amsterdam, De Boelelaan 1117, Amsterdam, Netherlands

^2^Cancer Center Amsterdam, Imaging and Biomarkers, Amsterdam, Netherlands

^3^Cancer Center Amsterdam, Department of Hematology, Amsterdam UMC, Vrije Universiteit Amsterdam, De Boelelaan 1117, Amsterdam, Netherlands

^4^Department of Internal Medicine, Amstelland Hospital, Amstelveen, The Netherlands

^5^Department of Epidemiology and Data Science, Amsterdam Public Health Research Institute, Amsterdam UMC, Vrije Universiteit Amsterdam, Amsterdam, The Netherlands

^6^Amsterdam Public Health research institute, Methodology, Amsterdam, The Netherland

**Corresponding Author**: Maria C. Ferrández

[m.c.ferrandezferrandez@amsterdamumc.nl](mailto:m.c.ferrandezferrandez@amsterdamumc.nl)

Amsterdam UMC, De Boelelaan 1117, 1118,

1081 HV Amsterdam, The Netherlands

Tel: +31(0)204449638

Fax: +31(0)204444329

**Funding:** Hanarth Fonds Fund and the Dutch Cancer Society (#VU-2018-11648)

**Supplementary Material 1**

ComBat harmonization was applied to align the probabilities from the three different reconstructions used in this study. The ComBat method assumes that the deviation introduced by the batch effect is removed once the means and the variances are standardized across the different batches. The value of the feature *Y* for a specific patient *j* and scanner *i* is expressed as follows:

$Y_{ij}= \alpha+ \gamma_{i}+\delta_{i}\varepsilon_{ij},$ (1)

where $\alpha$ represents the mean value of the feature *Y*, $\gamma$ represents the additive effect of the scanner, $\delta$ is the multiplicative effect of the scanner and $\varepsilon$ is the error. In this case, the feature *Y* would be the probability value and the VOI *j* the corresponding MIP. This harmonization method uses the empirical Bayes framework to estimate the batch/scanner effect terms, $\gamma_{i}$ and $\delta_{i}$. Subsequently, the corrected Y value ${Y_{ij}}^{ComBat}$ is calculated in equation (2) where$\hat{\alpha}$ ,$\hat{\gamma_{i}}$ and $\hat{\delta_{i}}$are estimations of parameters $\alpha$, $\gamma_{i}$ and$\delta_{i}$ respectively.

${Y_{ij}}^{ComBat}= \frac{Y_{ij}-\hat{\alpha}-\hat{\gamma_{i}}}{\hat{\delta_{i}}}+\hat{\alpha}$ (2)
